# Editorial: Innovative antibiofilm strategies: advancing the management of microbial biofilm infections

**DOI:** 10.3389/fmicb.2026.1944225

**Published:** 2026-08-06

**Authors:** Enea Gino Di Domenico, María Guembe, Giovanna Batoni

**Affiliations:** 1 UOSD Microbiology, San Gallicano Dermatological Institute, IRCCS, Rome, Italy; 2 Instituto de Investigación Sanitaria Gregorio Marañón, Madrid, Spain; 3 Department of Clinical Microbiology and Infectious Diseases, Hospital General Universitario Gregorio Marañón, Madrid, Spain; 4 Department of Translational Research and New Technologies in Medicine and Surgery, University of Pisa, Pisa, Italy

**Keywords:** antibiofilm, biofilm, catheter-associated infections, diagnostics, infection, quorum sensing

Microbial biofilms constitute a significant challenge in the treatment of infectious diseases. Their clinical significance arises from the development of a distinct multicellular lifestyle in which surface-associated microorganisms become spatially organized and enclosed within a protective extracellular matrix. This type of biofilm growth is characterized by metabolic heterogeneity, increased tolerance to environmental and therapeutic stresses, and limited accessibility to host immune clearance. As a result, biofilm formation can convert manageable infections into chronic, relapsing disorders, particularly in the context of indwelling medical devices, chronic wounds, and respiratory, oral, and urinary tract infections.

The Research Topic “*Innovative antibiofilm strategies: advancing the management of microbial biofilm infections*” is based on the following premise: effective antibiofilm therapies are unlikely to emerge from a single antimicrobial class. Rather, this significant advancement necessitates a mechanistic understanding of how biofilms are formed, sustained, sensed, destabilized, and ultimately controlled within the complex interplay of microbial communities, material interfaces, and host environments.

The articles presented here depict biofilms as dynamic, regulated systems that are vulnerable at specific molecular, metabolic, physical, and ecological nodes. Importantly, the Research Topic includes bacterial and fungal biofilms, acute and chronic infections, host tissues and abiotic devices, as well as interventions ranging from nutritional molecules and agricultural byproducts to phages, antimicrobial coatings, nanoparticles, extracellular vesicles, and electroceuticals. This breadth is not incidental. It represents the fact that biofilm-associated infection is a recurring phase of microbial structure that emerges when local ecology, surface chemistry, host immunity, and antimicrobial pressure all come together (Hall-Stoodley et al., 2004; Sauer et al., 2022).

A first group of contributions dissects the regulatory circuitry that enables biofilm formation and persistence. In *Vibrio parahaemolyticus*, Ni et al. define a feedback architecture in which the LysR-type regulator AcsS and the phosphodiesterase TpdA coordinate c-di-GMP levels, exopolysaccharide gene expression, and biofilm output. This study confirms the notion that biofilm formation is an actively regulated developmental program rather than a passive stress response by linking transcriptional regulation to second-messenger metabolism.

The same approach applies to Gram-positive pathogens. Yang et al. show that the molecular chaperone ClpB supports methicillin-resistant *Staphylococcus aureus* biofilm formation by sustaining extracellular matrix production and contributing to pathogenicity in a skin infection model. Kayser et al. extend this regulatory view to the ecology of the skin microbiota, finding that extracellular DnaK from *Staphylococcus epidermidis* can alter *S. aureus* biofilm formation and proteomic organization in a strain-dependent manner. Together, these studies place protein homeostasis and interspecies molecular communication at the center of biofilm biology, highlighting targets that are neither conventional resistance genes nor essential growth determinants, but rather modulators of community architecture and virulence expression.

Environmental control represents a second organizing theme. An et al. examine *Pseudomonas aeruginosa* from pulmonary infections and show that iron availability simultaneously enhances growth, adhesion, and biofilm formation while attenuating acute virulence phenotypes. This finding highlights an important principle: microbial persistence can be sustained by adaptive states that enhance colonization, tissue residence, and immune evasion while attenuating acute virulence. Similarly, Afsharnia et al. use transcriptomics to show that glucosamine, but not its acetylated derivative N-acetylglucosamine, inhibits *S. aureus* biofilm formation through broad metabolic rewiring, including effects on adhesion genes, arginine biosynthesis, the tricarboxylic acid cycle, urea metabolism, and pH homeostasis. These findings show that metabolism promotes biofilm formation and persistence. Targeting key metabolic pathways may therefore weaken biofilm structure and enhance the effectiveness of biofilm control strategies.

A third focus of this Research Topic concerns the development of experimental models that more accurately recapitulate biofilm-associated infections as they occur in clinical settings and on medical devices. Reese et al. present a modular dual-loop chemostat for multispecies biofilms on implant-relevant surfaces, with independently adjustable nutrient and flow conditions. By decoupling cultivation from hydrodynamic exposure, the system captures a neglected determinant of biofilm phenotype: shear stress. Their findings, which include flow-dependent shifts in biomass, robustness, matrix content, and species distribution on titanium surfaces, highlight the limitations of static monospecies assays while also providing a platform for testing antimicrobial coatings and implant designs under more realistic physical constraints. Such technologies are essential if the antibiofilm field is to improve its predictive power.

Verheul et al. also address this translational gap, examining whether the activity of phage-antibiotic combinations against planktonic *S. aureus* can be reliably predicted against mature biofilms on metal implant mimics. Their findings had strong translational implications, showing that planktonic screening was effective in identifying antagonistic interactions but had limited ability to predict synergistic effects after many treatments of mature biofilms. Thus, while rapid planktonic testing may help exclude harmful combinations, biofilm-specific models remain necessary to identify truly effective regimens. This distinction is particularly relevant as bacteriophage gain renewed clinical interest and combination design becomes increasingly empirical (Palma and Qi, 2024; Gallina et al., 2026).

Several contributions investigate antibiofilm approaches derived from natural, host-associated, or sustainable sources. For instance, Ma et al. show that stevioside, a natural sweetener, reduces *Streptococcus mutans* biofilm formation, acid production, bacterial viability, extracellular polysaccharide synthesis, and the expression of virulence-related genes, positioning it as an anti-cariogenic strategy for oral health. Sateriale et al. show that polyphenol-rich pomegranate peel extract from agri-food waste has antifungal activity against developing *Candida* pathogens by inhibiting membrane permeability, germ-tube formation, adhesion, and established biofilms. Rehman et al. add a host-derived dimension by showing that human urinary extracellular vesicle preparations inhibit biofilm formation by uropathogenic *Escherichia coli, P. aeruginosa*, and *Klebsiella pneumoniae* at physiologically relevant concentrations, without merely inhibiting planktonic growth. These studies converge on a key point: antibiofilm activity may result from agents that attenuate virulence, adhesion, or community formation rather than direct microbicidal pressure.

The review by Nakazwe et al. places these strategies within the broader framework of “resistance breakers,” which are drugs targeted to impair the processes that allow biofilms to tolerate antimicrobial exposure. These techniques, which target the matrix, efflux systems, enzymatic defenses, membrane permeability, quorum sensing, or biofilm metabolism, aim to restore antimicrobial efficacy by weakening the protective biofilm state. This concept is particularly relevant in light of the persistent limitations of the antibacterial pipeline and the biofilm capacity to compromise the effectiveness of available therapies (Ciofu et al., 2022; World Health Organization, 2024).

Engineered materials and nanotechnologies are another translational frontier. López-Lisbona et al. focus on tracheobronchial stents, a clinically important surface where bacterial colonization can complicate airway management. Their study identifies *P. aeruginosa* and *S. aureus* as frequent colonizers after stent placement and shows that silver-coated silicone markedly reduces the viability of clinical isolates on silicone surfaces. Tan et al. develop a redox-responsive ginsenoside-copper nanoagent that remains stable under physiological conditions but releases active components and generates reactive oxygen species in reduced environments, resulting in antibacterial and biofilm-disruptive effects against both Gram-positive and Gram-negative bacteria, with favorable *in vitro* biocompatibility. Collectively, these studies exemplify the ongoing transition from passive biomaterials to active anti-infective surfaces and responsive nanosystems (Balaure and Grumezescu, 2020; Su et al., 2025).

Host-directed and biophysical strategies further broaden the therapeutic landscape of antibiofilm research. Wilbanks et al. show that a DNABII-targeted monoclonal antibody induces biofilm collapse, which releases non-typeable *Haemophilus influenzae* cells in a form that neutrophils can recognize, promoting engulfment, NETosis, and immune-cell migration. These findings support the emerging concept that disrupting the matrix can shift bacteria from a protected immune-evasive state to one that is more vulnerable to host clearance (Fleming and Rumbaugh, 2018; Ciofu et al., 2022). Bhasme et al. review how electrical and redox cues influence wound repair and microbial behavior, including epithelial regeneration, immune responses, bacterial energetics, quorum sensing, and matrix organization. Together, these studies suggest that biofilm control is a system-level challenge requiring coordinated modulation of microbial, host, and environmental determinants that sustain persistence.

Across the studies in the Research Topic, a subtle but important methodological message emerges. Biofilm research must now move beyond whether an intervention reduces biomass and instead focus on the biological transition it induces within the biofilm ecosystem. Does it prevent initial attachment, weaken the matrix, force dispersal, sensitize persister-like cells, alter metabolic heterogeneity, restore immune access or reduce the risk of recurrence? Does it act similarly across strains, species and surfaces, or does it depend on local ecology? These questions matter because a reduction in crystal-violet staining, a decrease in colony counts, a shift in matrix composition and an increase in immune clearance are not interchangeable outcomes. The next generation of antibiofilm research will therefore require integrated readouts that connect structure, viability, function, host response and clinical feasibility.

Taken together, the contributions to this Research Topic reveal four priorities for the next phase of antibiofilm research. First, therapeutic discovery must target biofilm-specific physiology, including second-messenger signaling, protein homeostasis, matrix biosynthesis, metabolism and environmental sensing. Second, models must become more clinically faithful by incorporating multispecies communities, shear forces, implant surfaces, host effectors, and repeated treatment schedules. Third, antibiofilm strategies should be evaluated not only for biomass reduction or killing, but also for their ability to alter virulence, dispersal state, immune susceptibility, recurrence potential, and compatibility with existing antimicrobials. Fourth, translation will require integration: phages with antibiotics, coatings with diagnostics, natural products with mechanistic validation, host-derived factors with immunology, and electroceuticals with real-time sensing.

The field is entering a more ambitious stage. Biofilms are no longer seen only as barriers to drug penetration; they are ecosystems with regulatory logic, material properties, metabolic dependencies, and host interfaces. The clinical burden of biofilm-associated infections remains significant, but the conceptual and technological toolkit is expanding rapidly. By integrating mechanistic microbiology, biomaterials science, nanotechnology, natural-product research, host-pathogen biology, and bioelectronic medicine, this Research Topic captures the diversity of strategies now required to advance from *in vitro* antibiofilm activity to durable infection control in patients.
